# Chicago Medical Society

**Published:** 1887-09

**Authors:** 


### Society Reports.

### CHICAGO MEDICAL SOCIETY.

[Official Report.]

STATED MEETING, Tuesday, July 5, 1887.

The President, W. T. Belfield, M. D., in the chair.

Dr. E. B. Weston reported

### "CASES OF SYNOVITIS OF THE KNEE-JOINT."

Synovitis, though a surgical disease, is one which the general practitioner often treats—is often compelled to if he have a country practice—and there is no reason why he should not treat it well and successfully.

A slight synovitis may recover rapidly without treatment, and so these cases get well when treated with a poultice, or painted with tincture of iodine. But the physician who makes this his routine practice will not have many "flattering successes," and the number of cripples in his neighborhood may be monuments to his want of thoughtful attention.

I have four cases of synovitis of the knee to report. Not that they present points of peculiar interest, but to emphasize their importance, even though I disclose my own mistakes in doing so.

*Case 1.*—A. B., Irish, aged about 50, laborer. I met him on the street, January 1, 1885, when he told me he had pain in his left knee; that he did not know of injuring it in any way. Having a physician's dislike for using "all out doors" for his office, and being consulted wherever he chances to meet a would-be patient, I gave him little attention and advice of a very general nature. One week later he sent for me. I found him in bed suffering severe pain, knee distended with fluid in joint cavity, leg flexed. He refused to have fluid evacuated, or even to have leg put on splint, although I plainly told him of the probable consequences of not doing so. Then, instead of leaving the case, I allowed him to be his own physician, while I continued to make daily visits and suggestions.

I have no full notes of the case, but it went from bad to worse. Abscesses formed in the thigh, which were opened as they developed; drainage tubes passed in various directions, the limb becoming honey-combed by the burrowing pus. These cavities were frequently syringed with carbolized water and the limb kept on a pillow of oakum. He was given a nourishing diet, stimulants and tonics. But he gradually lost flesh and strength, temperature increased, and on the 1st of March he died of sepsis and exhaustion.

*Case 2.*—C. D., American, aged about 45, by profession a lawyer. First saw him March 4, 1886, when he told me his left knee had pained him quite constantly, but not very severely, for about ten days. He thought he had taken cold in it while sitting at his desk, under which he had felt a draught. I found knee moderately enlarged, joint distended with fluid and hot and painful. I ordered him to bed and had applied an anodyne liniment and warm fomentation.

This treatment was continued for three days without benefit, when I opened the synovial cavity with a bistoury and evacuated about eight ounces of light straw-colored, probably nearly normal, synovial fluid. He was at once relieved of pain, the limb put on a well-padded posterior splint, and an elastic bandage applied. The fluid gradually re-accumulated, and in four days the joint was again full, hot and painful. I now aspirated it and re-applied bandage. There was again a slight filling, but not sufficient to necessitate opening. The patient steadily improved, and in a few weeks had perfect use of the joint.

*Case 3.*—E. F., a Swede, aged 25, painter. February 19, 1886, at noon, while on his way to work he fell, striking his right knee on the ice. It caused him severe pain for a short time, but he worked the whole afternoon, standing on a step-ladder, without much discomfort. At night the knee began to swell and pain him intensely. When I saw him the knee was hot, very painful and moderately distended, and leg flexed. A liniment composed of opium, aconite and menthol was applied, with warm fomentation, and opium given internally. No improvement taking place, and the joint becoming much distended, on the 22nd inst. I opened it with a bistoury, letting out a large quantity of blood-colored fluid. He was at once relieved of his intense pain. He made a steady recovery, and in about two months had perfect use of the joint.

*Case 4.*—S. H., aged 38, a carpenter. He had been at work on outside of building most of the winter, and of late had been obliged to kneel on scaffolding a good deal. When I saw him on February 3d, he told me his left knee had been painful and getting stiff for sometime. It was then painful and contained a small amount of fluid. I could not convince him of the importance of giving it rest. I saw him occasionally during the following weeks, during which he was working about and simply using a liniment. On the 11th instant, the condition of the knee being decidedly worse, I told him unless he was willing to give me absolute control of the case, I should do nothing more for him. He was unwilling to submit, and I did not see him again for four days, when he sent for me. The joint was now moderately distended, and the patella floating. I applied posterior splint and elastic bandage, and the patient went to bed. Three days later, the joint being very full, I opened it with a bistoury, letting out a quantity of apparently unchanged synovial fluid. Immediate relief followed, and the bandage was re-applied. The patient was given quinine, iodide of iron, and the kidneys and bowels kept active. But the improvement was not permanent. The joint slowly re-filled, and in three weeks it became necessary to evacuate it. This time I used the aspirator, and an accident happened which I am quite sure will not occur to me again. Being ready to introduce the needle, I asked my friend, who was assisting—a most careful and intelligent physician—to exhaust the aspirator bottle. All being ready, I passed the needle into the tissues until the opening near the joint disappeared, when I requested my assistant to turn the stop-cock, and as he did so I thrust the needle into the cavity of the joint. I was startled by a shriek of pain, and I have rarely seen such an agonized expression on any face. The muscles of the leg instantly became rigid and prominent, and the joint increased in size, but no fluid appeared. Fortunately the probable cause of the trouble at once occurred to me. The bottle had not been exhausted, but filled with compressed air. The needle was withdrawn, the aspirator made ready and the fluid evacuated as intended. The air which had been driven into the joint bubbled out with the liquid. But the cellular tissue of the thigh contained air which could be felt for two weeks, by which time it had disappeared, leaving no ill effects. Apparently the joint was not injured by its forcible distension, though in four days it had become filled again, and I withdrew with the aspirator fluid of the same appearance as before. In just one week, the other knee having become similarly involved, and the joint cavity very full, I aspirated it. From this time he steadily improved and made a perfect recovery.

I have intentionally omitted to speak of the anatomy of the knee, or of the pathology, diagnosis and classification of the cases I have briefly reported, but it would seem proper to treat them by pressure, absolute rest, and early, and if necessary repeated evacuations. Barwell, whose reputation in diseases of the joints is certainly second to no man's, says: "If the inflammation be sufficient to cause considerable secretion into the joint, producing marked fullness of the sac, the synovial membrane should be punctured . . . to relieve the tension."

Howard Marsh, in his work on "Diseases of the Joints," says: "It is now well known that matter, whether connected with acute or chronic arthritis, may be safely evacuated, with the result that the severe suffering, the prolonged fever, the wide and destructive burrowing, and the formation of sinuses, which were the common rule only a few years ago, can be generally avoided."

Dr. G. J. Tobias:

I think the paper is a very good one and the cases reported interesting. The doctor states that in two cases he made an incision before he aspirated. I would ask why he did it, why not try the aspirator first? The accident spoken of has occurred before in this city, that of compressing the air in the bottle instead of pumping it out and producing a vacuum, thereby causing compressed air to be forced into the joint cavity, with sometimes most serious results. Would it not be a good plan to have some basin or small receptacle at hand, containing water, to try the aspirator first and see that it is in proper working order? I wish he would also tell us more fully how he used the posterior splint and whether he padded it, the material used for bandage, etc.

The President:

I take it for granted that Dr. Weston took the ordinary cleanly and antiseptic precautions before incising the knee-joint. Most of us would be inclined to use the aspirator before making an incision, since it is a common observation that after the knee-joint has been aspirated once, twice, three times or more, resolution has occurred and the functions of the joint resumed even when the fluid was purulent. The accident in the use of the aspirator reminds me of one of which I am personally aware as occurring in this city, and which had a more unfortunate termination than in Dr. Weston's case, where most of the air got into the tissue outside of the joint. In the case to which I refer the stop-cock was not turned until the needle had entered the joint, and in three minutes the patient was dead.

Dr. E. B. Weston, in closing the discussion, said:

I used the bistoury instead of the aspirator because it was more convenient at that time. The bistoury has been used a good many times, but I don't pretend to say it is better, perhaps not so good, but in my case it was a matter of convenience. The cases did well. The posterior splint was well padded with cotton. I used for the splint heavy sole leather. An elastic bandage, or rather a flannel bandage, was used below the knee and on the thigh and a rubber bandage to make the pressure over the knee-joint. Of course the aspirator should have been tested. The mistake should not have occurred, and yet no injury was done by the violent distension of the joint, or by the air which entered it.

Dr. William T. Belfield reported

A CASE OF MERCURIAL NECROSIS WITH SPECIMEN.

The case of mercurial necrosis that I wish to mention is that of a young man, apparently in excellent health, who seven years ago contracted a chancre which was followed by the usual symptoms of constitutional syhpilis. He was treated by a regular physician in an Eastern city, and for a year and a haif had the usual history of a healthy man contracting syphilis—mucous patches, occasional rashes on the skin, etc. After about two and a half years, during which time he was under constant observation and much of the time taking medicine, he had no further indication of constitutional syphilis. It went along that way for about four years. Over six years after the contraction of the chancre, he noticed a slight lump on the instep of the left foot which gave a little pain when he wore tight shoes. Fearing this might be an indication of the return of the disease, he went to his physician who prescribed syphilitic treatment. He ordered the young man to take one-fourth grain of yellow oxide of mercury pill three times a day. The patient, who was naturally anxious to overcome any taint that might remain in him, followed the direction faithfully, and for six months took one of these pills three times a day. At the end of this time his mouth became sore. About ten days after he first noticed the soreness of the mouth, being then in Chicago, he was directed to see me. At that time his gums were quite soft and spongy, his teeth felt loose and he could not bite bread with any comfort. On the left side the alveolar process of the upper jaw was exposed for about three-fourths of an inch in length and one-half inch in vertical breadth. The three molar teeth which were fixed in that portion of the jaw were decidedly loose. The hard palate corresponding to that part of the alveolar process was swollen, and there were the usual symptoms of subacute periostitis. He was merely ordered to discontinue the mercury, use a simple mouth-wash and take iodide of potassium. In the course of ten days the portion of the jaw exposed was so loose that it could have been removed without difficulty, but the patient, flinching from the slight pain this would cause, declined to have it removed and worked at it with his fingers until, about three weeks after I first saw him, he pulled the piece away. In the meantime he had lost three sound molar teeth. The piece is quite small, merely large enough for the insertion of the three teeth, but it is of some interest. The upper surface of it is the floor of the antrum. During a part of the time the cheek-bone was swollen and tender, but no abscess formed.

It may be questioned whether this necrosis was mercurial or syphilitic. The man unquestionably, seven years ago, had syphilis, but a little consideration shows that this necrosis was not syphilitic. In the first place, for four and a half years he has had no indication of syphilis; this little hardening of the instep, for which he took the mercury, still persists and is evidently not of syphilitic origin; furthermore, there was thickening and softening of the gums and loosening of the teeth, which was due to the mercury; and, lastly, while syphilis may cause necrosis of the long bones and of many other bones in the body, yet it rarely causes necrosis of the inferior and superior maxillary, while mercury causes necrosis in these bones with especial frequency.

Dr. Belfield said, in reply to a question, that the patient had not been salivated during the time of treatment, so far as could be remembered.

Dr. Joseph Haven exhibited

### A MECHANICAL SUPPORT FOR MAINTAINING THE LITHOTOMY POSITION.

The position most favorable for the majority of the operations on or about the perineum is that which is known as the lithotomy position—the patient prone upon the back, thighs sharply flexed on the abdomen, knees separated. For the patient to maintain this position unaided is a difficult procedure, and when under an anæsthetic an absolute impossibility. Hence the necessity for assistance in some form. The usual custom is to entrust each limb to an assistant to hold during the operation. To this procedure there are some objections:

First, it increases the necessary number of assistants by at least two. Usually the patient, if not the whole household, is dreading the operation with nervous anxiety, and the sight of a number of assistants, or an unnecessary display of paraphernalia, does not tend to quiet their fears or relieve their anxiety; hence operations at the house should be conducted with as little display as possible.

Again, any one who has assisted in holding a patient in the lithotomy position for an hour or so knows that it is a tiresome undertaking.

Again, emergencies are continually arising where they are called upon to render other assistance, as from their proximity to the operator they are in the way of others, and are called upon to pass instruments, wash sponges, etc., thereby neglecting their *own work* and the falling of the patient's foot in the operator's lap does not tend to *better* the results of the operation or improve the temper of the operator. Consequently it is not strange that various appliances should have been improvised by which the patient could be held in the proper position.

My attention was first called to this subject some six or eight years ago. The operation was for the closing of a lacerated cervix. The operator brought with him an apparatus made of three boards, about four feet long, joined on the sides by hinges. When the side-pieces were raised and fastened by hooks, the whole formed a sort of trough in which the patient was placed and a harness arrangement bound each limb to the corresponding side-piece. While the apparatus worked well upon this occasion, there are still objections to it.

In the first place, it required too long a time to extricate the patient from the apparatus.

Secondly, the effect of putting together such a contrivance in the sick-room might be similar to the building of a scaffold in the presence of the condemned.

Thirdly, blood-stains are not easily removed from wood, and should there be any septic influence in the case, this would be readily absorbed in the pores of the wood; and I doubt the advisability of using a wooden apparatus in repeated operations in close proximity to open wounds.

In a work entitled "Minor Surgical Gynæcology," by Paul Mundé, the author speaks favorably of an apparatus designed by Dr. Hamilton, of Harrisburg, Pa., which he styles "Hamilton's Gynapod." This consists of a piece of wood upon which the buttocks rest; from this arises on either side an arm surmounted by a crutch which supports the knee. While being more simple than the apparatus previously described, some of the same objections hold good in this case. Again, as the limbs are not fastened in any way, it allows too much freedom to the feet. This will be particularly annoying if the operation be a trivial one, not requiring the administration of an anæsthetic.

If, then, an apparatus for this purpose is of service in these operations, what should be the essential requirements?

First: It should be so constructed that it can be instantly removed if emergency should arise.

Second: It should be of a material that will admit of its being thoroughly cleansed and disinfected.

Third: It should secure the patient firmly and at the same time be as simple, light and easy of transportation as possible.

Acting upon these thoughts I had made, two years ago, an apparatus based upon the same principle as the one which I shall show you this evening, namely, a light nickel-plated bar provided with a hinge in the center, allowing it to be folded up when not in use. This bar passes under the knees of the patient, holding them flexed on the abdomen by means of a band of webbing which passes around the shoulders of the patient, the webbing having a strap and buckle allowing the band to lengthen or shorten according to the size of the patient. Bands of the same material at either end of the rod securely hold the limbs below the knee, every strap being attached to the rod by means of a snap-catch, which can be instantly disengaged if occasion so demands. A rod attachment for holding a fountain syringe completes the outfit. The whole apparatus, when not in use, fits into a case eighteen inches long, five inches deep and four inches wide.

In this connection it becomes my duty, and at the same time a pleasure, to accord to Dr. Thomas B. McBride, of Philadelphia, the credit of having, within the last few months, devised an apparatus somewhat similar in principle to the one just described. A description of his apparatus will be found in the *Medical and Surgical Reporter* for May. He writes me that he has, since that publication, added the hinge, thereby lessening the length of the apparatus when not in use.

In conclusion I will add that I have found the apparatus serviceable in all operations where the lithotomy position is indicated, and particularly when operating for slight lacerations of the cervix where no anæsthetic was administered.

The President asked Dr. Haven whether the apparatus answered as well when the patient was under an anæsthetic.

Dr. Joseph Haven:

It answers better. I have used the apparatus a number of times with an anæsthetic and several other physicians have used it. The patient is liable to come out of either before the operation is finished, and with this apparatus they cannot do themselves any harm during the operation.

Dr. Tobias asked Dr. Haven in how many cases of laceration of the cervix he had tried the apparatus, and if the patient ever raised any objection to its use.

Dr. Haven :

Probably twelve or fifteen. No objection has been made to to the apparatus except in one case; but you will find that some patients object to any instrument. This patient asked me what it was, and when I explained that it was not an instrument of torture, she readily consented to its use. However, where an anæsthetic is to be given I do not usually put it on until the patient is anæsthetized, and they do not see the instrument at all. It can be put on in less than one minute and taken off in the same time. I very often use it in dressing wounds around the uterus, packing the vagina after opening a pelvic abscess where I do not care to employ an assistant to hold the patient in the lithotomy position, or for slight operations on the rectum—anything that would not require an anæsthetic.

